# Vitamin C in Allergy Mechanisms and for Managing Allergic Diseases: A Narrative Review

**DOI:** 10.3390/children12060718

**Published:** 2025-05-30

**Authors:** Chiara Trincianti, Matteo Naso, Maria Angela Tosca, Giorgio Ciprandi

**Affiliations:** 1 Allergy Center, IRCCS Istituto Giannina Gaslini, 16100 Genoa, Italy; chiaratrincianti@gaslini.org (C.T.); matteonaso@gaslini.org (M.N.); mariangelatosca@ospedale-gaslini.ge.it (M.A.T.); 2 Allergy Clinic, Casa di Cura Villa Montallegro, 16100 Genoa, Italy

**Keywords:** vitamin C, oxidative stress, type 2 inflammation, allergy, immune modulation

## Abstract

Allergic diseases share a type 2 immune reaction and elevated oxidative stress, contributing to disease pathogenesis and exacerbations. Vitamin C (ascorbic acid), a fundamental exogenous antioxidant, has been hypothesized to attenuate these pathological mechanisms. This narrative review critically examined the most recent evidence concerning the role of vitamin C in preventing and managing allergic diseases, including asthma, allergic rhinitis, and atopic dermatitis. This narrative review consisted of three steps: conducting the search, reviewing abstracts and full texts, and discussing results. For this reason, we consulted the PubMed database to detect the pertinence of studies according to the review’s conduct. The final search ended in March 2025 and included English-language-based international articles, online reports, and electronic books. The keywords “vitamin C and allergic disease” and “vitamin C and immune system” were used. After the complete search, we read the abstracts to ensure that they concerned the topic of interest. Recent evidence suggests a protective role for vitamin C in asthma, with several studies reporting reduced oxidative stress markers, improved lung function, and decreased airway inflammation following regular intake or supplementation. Higher dietary vitamin C intake correlates with lower asthma prevalence and severity, particularly in pediatric populations. Conversely, the findings regarding allergic rhinitis and atopic dermatitis are heterogeneous. While topical ascorbic acid derivatives show promise in atopic dermatitis models, oral vitamin C intake does not appear to affect allergic rhinitis or dermatitis risk significantly. Vitamin C demonstrates potential as an add-on therapy in asthma management by attenuating oxidative stress and type 2 respiratory inflammation. However, its role in allergic rhinitis and atopic dermatitis remains less clear. Further multicentric, well-designed clinical trials are necessary to establish definitive guidelines for vitamin C supplementation in allergic disease management.

## 1. Introduction

Ascorbic acid (ASC) is an essential human vitamin, as the body cannot produce it. Also known as Vitamin C, ASC is a diet-derived exogenous antioxidant that protects against lipid peroxidation and, consequently, oxidative stress [1]. It was discovered in the early 20th century by Albert Szent-Györgyi and identified as the essential nutrient that prevents scurvy, a disease that had plagued sailors for centuries.

Humans depend entirely on dietary sources to maintain their body pool of vitamin C. The body cannot synthesize it as it lacks the enzyme L-gluconolactone oxidase, which catalyzes the final step of ASC biosynthesis by oxidizing L-gluconolactone [2].

Fruits and vegetables are rich in ASC, in particular fresh fruits such as citrus fruits (oranges, lemons, and grapefruits), strawberries, kiwis, and guavas, as well as vegetables like bell peppers, broccoli, Brussels sprouts, and leafy greens such as kale and spinach [3]. However, the Kakadu plum, a fruit native to Australia and camu camu (Amazon rainforest) are among the richest sources of vitamin C, with around 5300 mg and 2000–3000 mg per 100 g, respectively—far more than citrus fruits, which contain about 50 mg per 100 g [4,5]. Acerola, native to Central and South America, is another powerful source, providing around 1500–1700 mg per 100 g [6].

Vitamin C exists in two primary forms in the body: the reduced form (ASC) and the oxidized form, dehydroascorbic acid (DHA), with ASC being predominant. Vitamin C absorption involves three mechanisms of membrane transport, such as passive diffusion, facilitated diffusion, and active transport. Passive diffusion is slow due to vitamin C’s anionic and water-soluble nature at neutral pH. However, passive diffusion may be more significant in acidic environments like the stomach or the small intestine, where more unionized ASC exists. Facilitated diffusion relies on carrier proteins, with DHA using glucose transporters to compete with glucose for absorption. Active transport, mainly through the sodium-dependent vitamin C transporter family (SVCT), identified by Tsukaguchi et al., plays a significant role in ASC absorption in the intestine [7]. They later demonstrated that the intestine utilizes SVCT1, a low-affinity, high-capacity active transporter. Studies have also shown that as oral doses of ASC increase, its absorption efficiency decreases, leading researchers to conclude that SVCT1 is saturable and dose-dependent [8]. Indeed, vitamin C steady-state plasma concentrations followed a sigmoid kinetic pattern concerning dosage. The steepest increase occurred between daily doses of 30 mg and 100 mg. The first dose exceeding the sigmoid phase was 200 mg daily, while complete plasma saturation was reached at 1000 mg per day [8]. Levine et al. proved that Vitamin C bioavailability was 100% after a single oral dose of 200 mg but decreased to −33% after a single dose of 1250 mg [9].

Although only about 10 mg of Vitamin C daily is needed to prevent scurvy, the recommended daily intake is much higher, up to 100 times greater than many other vitamins. Since the body has limited storage for this water-soluble vitamin, regular intake is necessary to avoid deficiency [10]. Plasma levels of the vitamin fluctuate with age, with the highest in 6- to 11-year-old children and with sex, being higher in women than in men [11]. The recommended dietary allowance (RDA) for vitamin C varies by country, with the United States and Canada recommending 90 mg/day for men and 75 mg/day for women. Italy suggests 105 mg/day for men and 85 mg/day for women. Males generally require more vitamin C than females due to their higher body weight and fat-free mass. RDAs for children and adolescents are based on adult needs but adjusted for their lower body mass. In Italy, for example, the Italian Society of Human Nutrition (SINU) recommends an intake of 45 mg for children from 4 to 6 years of age, while elderly individuals may need a higher vitamin C intake, indicating a daily intake of 120 mg for subjects from 75 years of age [12,13].

Allergic diseases are characterized by a type 2 inflammation associated with oxidative stress [14]. In this regard, vitamin C could play an interesting role in dampening allergic inflammation and reducing oxidative stress.

Asthma is a heterogeneous chronic inflammatory disorder of the lower airways that affects nearly 400 million people worldwide [15]. It is the most frequent chronic disease in children [16]. It is defined by variable airway obstruction and bronchial hyperresponsiveness. Children with asthma could have shortness of breath, recurrent episodes of wheeze, cough and/or chest tightness. Its pathogenesis is not completely understood due to heterogenic gene-environment interactions [17]. Chronic lower airway inflammation is known to be more common in individuals that also have inflammatory disorders of the upper airway, like allergic rhinitis (AR). Allergic rhinitis is an atopic disease characterized by sneezing, rhinorrhea, and/or nasal congestion/obstruction [18]. These symptoms must be related to an allergen exposure that induces an immunoglobulin E (IgE)-mediated type 1 hypersensitivity response. The prevalence of AR has been reported from 5% to 50% worldwide, increasing across the globe proportionally with age until young adulthood [19]. Similarly, atopic dermatitis (AD) is a common inflammatory disorder of the skin. Patients with AD have recurrent itchy rashes, dry skin, and eczema, which significantly impact their quality of life and affect the lives of their families [20]. The prevalence of AD in children is approximately 20%, ranging between 7 and 14% in adults [21].

This narrative review aimed to present and discuss the most recent studies (the last five years) on vitamin C and allergic diseases with a focus on AR, asthma, and AD.

## 2. Methods

This narrative review followed three steps: conducting the search, reviewing abstracts and full texts, and discussing the results. For this purpose, we consulted the PubMed database to detect the pertinence of studies according to the review’s conduct. The final search was conducted in March 2025 and included English-language-based international articles, online reports, and electronic books. The keywords “vitamin C and asthma”, “vitamin C and allergic rhinitis”, “vitamin C and atopic dermatitis”, and “vitamin C and allergy” were used, selecting a total of 255 articles. After the complete search, we analyzed the abstracts to ensure that they were consistent with the matter of the review. We removed all duplicates and reviewed the remaining abstracts to ensure that they fulfilled the review inclusion criteria. The eligible criteria included studies investigating vitamin C in at least one of the three aspects (in vitro, animal, and human). Therefore, these studies of interest were summarized and synthesized to integrate the narrative review. Since it is a narrative review, it was unnecessary to document the literature search on specific platforms.

## 3. Vitamin C Mechanisms of Action

Vitamin C is a water-soluble vitamin derived from glucose that carries out its antioxidant activity through several different mechanisms: it suppresses macrophage secretion of superoxide anions, acts as a free radical scavenger [22], downregulates the expression of genes encoding for pro-inflammatory cytokines (TNF-α, IL-1β, IL-6, IFN-γ), inhibits nuclear factor-kappa B (NF-κB) activation, promotes the production of the anti-inflammatory cytokine IL-10, and enhances the activity of anti-inflammatory enzymes like heme-oxygenase-1 (HO-1) [23]. Vitamin C is a potent antioxidant that helps combat oxidative stress by donating or transferring electrons [24]. It neutralizes harmful radicals like oxygen, nitrogen, and sulfur and helps regenerate other antioxidants, such as glutathione and Vitamin E [25]. In particular, oxidative stress due to ROS formation could act in vivo inducing the oxidative modification of low-density lipoproteins (LDL). In this case, α-tocopherol is the most abundant and important antioxidant and ascorbic acid could act recovering α-tocopherol [26]. Indeed, leukocytes, such as neutrophils and monocytes, actively accumulate vitamin C up to 50–100 times higher than plasma levels. Neutrophils reach maximum concentration with a dietary intake of approximately 100 mg/day and use the SVCT2 transporter to accumulate vitamin C, reaching at least 1 mM intracellularly, up to 10 mM when activated. The high vitamin C concentration in neutrophils is essential for protecting them from oxidative stress, as it neutralizes reactive oxygen species and regenerates other cellular antioxidants. An imbalance between oxidant production and antioxidant defenses can affect cellular signaling, activating the NF-κB transcription factor, which amplifies inflammation. Vitamin C can modulate immune function by reducing NF-κB activation and regulating intracellular redox signaling. Additionally, it protects neutrophil cell membranes from oxidation, improving their motility and function.

Studies on peripheral blood lymphocytes have shown how the pro-inflammatory cytokines TNF-α and IFN-γ decreased in incubation with vitamin C, while anti-inflammatory IL-10 production increased. Similarly, ASC addition to peripheral blood monocytes from pneumonia patients decreased the generation of IL-6 and TNF-α cytokines [10].

Vitamin C has also been shown to improve neutrophil migration in response to chemoattractants, enhance the phagocytosis of microbes, and stimulate the generation of reactive oxygen species (ROS) and the killing of microbes, thanks to a modulatory effect of nitric oxide (NO), whose synthesis is potentiated by ASC [27].

In vitro studies show that vitamin C can promote lymphocyte proliferation, increase antibody production, and make them more resistant to cell death. Additionally, it appears to be involved in the differentiation and maturation of immature T cells and natural killer cells.

Vitamin C deficiency increases susceptibility to infections, while evidence supporting its use in preventing viral diseases in well-nourished populations is limited. However, recent studies suggest that intravenous vitamin C may benefit patients with severe medical conditions like sepsis and acute respiratory distress syndrome (ARDS), potentially improving outcomes in critical illnesses such as COVID-19, with ongoing clinical trials to explore its effectiveness [12].

In addition, ASC improves the absorption of iron, calcium, and folic acid [28]. In particular, it enhances the bioavailability of non-heme iron, facilitating its absorption from dietary sources. Research suggests that vitamin C promotes iron reduction, improves its uptake and utilization, and helps regulate iron metabolism, which is essential for the proper functioning of the immune system [29].

**Figure 1:**
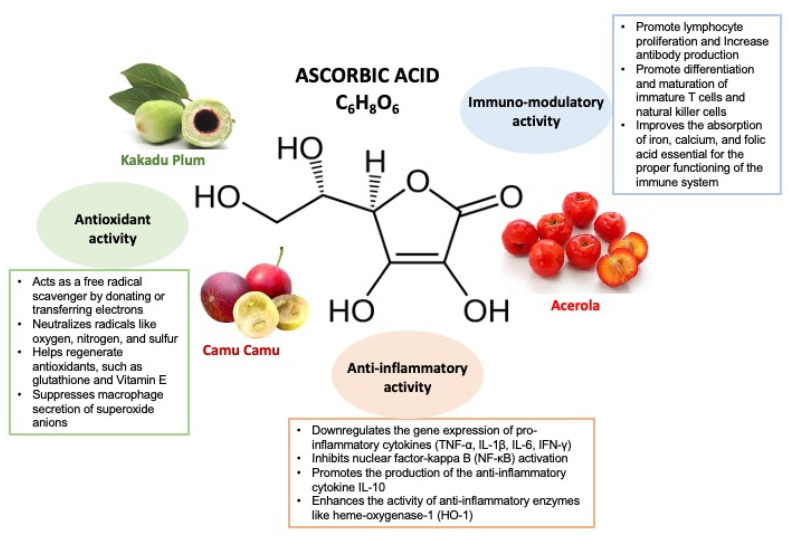
The main mechanisms of action of Vitamin C (ascorbic acid).

Figure 1, Table 1 and Table 2 summarize the main mechanisms of action and the reported studies.

## 4. The Inflammatory Basis of Allergic Diseases

Allergic diseases, such as respiratory and food allergies, result from an imbalance in the immune system, characterized by type 2 inflammation and excessive Th2 polarization. In allergic individuals, dendritic cells drive naïve T cells toward a Th2 phenotype, increasing IL-4, IL-5, and IL-13 production. These cytokines promote IgE synthesis, eosinophil recruitment, and mucus overproduction, fueling persistent inflammation. At the same time, a weakened type 1 immune response, with reduced IFN-γ levels and impaired Th1 activity, compromises immune regulation and defense against pathogens, further amplifying allergic inflammation [42,43].

In individuals with allergies, inflammatory cells generate significantly higher levels of reactive oxygen and nitrogen species (ROS/RNS) than healthy individuals. The excessive production of ROS is triggered by exposure to airborne allergens (such as ultrafine carbon particles, diesel exhaust particles, cigarette smoke, and pollen) and food allergens [44].

In particular, oxidative stress plays a crucial role in the pathogenesis and worsening of asthma. In asthmatic patients, mast cells, eosinophils, and macrophages produce high amounts of ROS through the respiratory burst, activated by the NADPH oxidase (NOX) enzyme in response to inhaled allergens. ROS activates pro-inflammatory pathways (MAPK, NF-κB, AP-1), increasing cellular damage and the production of inflammatory mediators, thereby exacerbating bronchial hyperreactivity [45,46].

In addition, IL-4 and IL-13 activate the enzyme inducible nitric oxide synthase (iNOS), significantly increasing nitric oxide (NO) production. NO is a signaling molecule produced by immune and epithelial cells, including respiratory epithelial cells [47]. It acts as a bronchodilator in the lungs and as a vasodilator, increasing plasma exudation [48]. For these reasons, fractional exhaled nitric oxide (FeNO) represents a non-invasive biomarker of type 2 respiratory inflammation that has been extensively studied in recent years [49].

## 5. Allergic Rhinitis

The effects of vitamin C on the immune system, specifically its influence on the development and control of different allergic diseases like AR, have been studied for several years.

First, Fortner et al. used the prick test method to examine whether ASC affects nasal responses in patients with AR or histamine skin reactions [38]. They tested eight adults with AR in a randomized, double-blind, placebo-controlled trial, administering 2 g/day of ASC or placebo (lactose placebo capsules) for 4 days. No difference between the two treatments was noted in the nasal response to the instillation of allergens. Similarly, higher doses of vitamin C (up to 4 g/day) did not reliably modify skin test results by the prick method with histamine.

In a 2004 study, the serum levels of dietary antioxidants, including ASC, showed no strong or consistent associations with the occurrence of aeroallergen sensitization [50].

Another interesting study conducted in 2005 collected data on the dietary and lifestyle habits of 1050 people aged 13–81, obtaining 568 blood samples for further analysis of total IgE, carotenoids, tocopherols, and vitamin C [51]. While plasma total carotenoids negatively correlated with the prevalence of allergic rhinitis, plasma vitamin C did not correlate with AR or allergic sensitization.

In another study published in 2018, 27 patients with (AR) were randomly assigned to one of three groups: a control group, an exercise group (walking or running for 30 min per session, three times per week for eight weeks), and a group combining exercise with vitamin C supplementation (2 g per day) [39]. The study concluded that aerobic exercise significantly improves AR symptoms (assessed using the Total Nasal Symptom Score (TNSS) questionnaire) and increases peak aerobic capacity and peak nasal inspiratory flow (PNIF). Still, the added benefit of vitamin C remains unclear.

## 6. Asthma and Ascorbic Acid Dietary Intake

Although studies regarding the implications of vitamin C in AR may seem unpromising, further research with larger studies is needed to clarify this issue. Contrarily, the studies conducted on the wheezing and asthma models are more encouraging.

For example, a study explored the relationship between consuming fruit rich in vitamin C and respiratory symptoms in children, focusing on wheezing and asthma [30]. This study included 18,737 Italian children aged 6–7 years. The findings showed that higher fruit consumption (5–7 times per week) significantly correlated with lower risk of wheezing and other respiratory symptoms, such as shortness of breath, chronic cough, and rhinitis. Even lower levels of fruit intake (1–2 times per week) offered some protective effects. This finding was especially noticeable in children with a history of asthma.

Promising results also emerged from a recent Chinese study that analyzed data from the National Health and Nutrition Examination Survey (NHANES) database [31]. The study examined the relationship between the dietary intake of various vitamins and their supplements (including vitamins A, C, D, E, B1, B2, B6, B12, K, niacin, folic acid, and choline) and childhood asthma. They concluded that the probability of asthma in children diminished with elevated vitamin C intake. Based on these findings, they recommended that adolescents increase their consumption of vitamin C-rich foods.

Other studies focused on preschoolers. A study showed how high dietary intakes of vitamins C and E may be associated with a reduced prevalence of asthma [52].

Another study demonstrated how childhood asthma could be related to significant reductions in enzymatic and nonenzymatic antioxidant defenses, highlighting oxidative stress as a key factor in asthma pathophysiology [53]. This study involved 164 children with mild asthma (ages 6–16) and 173 healthy controls. The included asthmatic children had no recent respiratory infections or asthma flare-ups. Tests included spirometry, total IgE measurement, eosinophil counts, and skin prick tests. Blood samples were analyzed for malondialdehyde, antioxidant vitamins (ASC, α-tocopherol, β-carotene, lycopene), enzymatic antioxidants (glutathione peroxidase, superoxide dismutase), and amino acids using High-Performance Liquid Chromatography (HPLC), enzyme-linked immunosorbent assay (ELISA) and gas chromatography in both groups. The results showed that children with asthma had significantly higher malondialdehyde levels, indicating greater oxidative stress (*p* < 0.001). In contrast, both nonenzymatic (glutathione, ASC, α-tocopherol, lycopene, β-carotene) and enzymatic antioxidants (superoxide dismutase, glutathione peroxidase) levels were significantly lower in children with asthma compared to healthy subjects (*p* < 0.001). Additionally, key amino acids involved in glutathione synthesis (glycine and glutamic acid) were significantly reduced in asthmatic children (*p* < 0.001), and some amino acids vulnerable to oxidative stress (histidine, proline, lysine, etc.) were also lower in asthmatic children, though not all differences were statistically significant.

Vitamin C deficiency is common in children with asthma and seems to be associated with increased asthma severity and reduced pulmonary function [32]. Indeed, a cross-sectional study published in 2022 collected data from 76 asthmatic children aged 7–17 years. In this group of patients, researchers measured plasma 8-iso-prostaglandin F2α (PGF2α) concentrations, a marker of oxidative stress formed by the lipid peroxidation of arachidonic acids (AAs). They also assessed plasma levels of zinc, vitamin C, and vitamin E, as well as pulmonary function tests using a spirometer, including forced expiratory volume in one second (FEV_1_) and forced vital capacity (FVC). A total of 72 participants exhibited high oxidative stress. All enrolled patients had zinc deficiency, and nearly 40% of them were deficient in vitamin C, a condition that appeared to be linked to more severe asthma and airway obstruction. Additionally, plasma zinc levels positively correlated with FEV_1_ and the FEV_1_/FVC ratio.

Similarly, a study recruiting 6293 adults confirmed that higher vitamin C intake was inversely associated with asthma prevalence [33]. Specifically, a higher intake of vitamin C (≥75 mg) was significantly associated with a lower prevalence of asthma in participants with high serum high-sensitivity C-reactive protein (hs-CRP) levels, a marker of inflammation; no such association was observed for allergic rhinitis.

Another recently published study reported conflicting results. It found no consistent evidence linking fruit and vegetable intake with asthma or chronic rhinosinusitis (CRS) among 3206 European adult participants. Of these, 22.8% reported asthma symptoms, and 19.5% had CRS [34].

The seemingly stronger association between vitamin C levels and bronchial symptoms may be attributed to the high concentration of ASC in lung tissue. ASC levels in alveolar macrophages and alveolar type II cells are approximately 30 times higher than in the bloodstream [54]. This fact suggests that vitamin C could play a protective role against oxidative stress in the lungs, facilitated by transport mechanisms such as glucose transporters (GLUT2) and sodium-ascorbate cotransporters (SVCT2), which have been identified in the human bronchial epithelium as key regulators of vitamin C levels in respiratory tract lining fluid [55].

Circulating vitamin C levels have not been linked to adult asthma risk. A 2024 study analyzed data from the National Health and Nutrition Examination Survey (NHANES) 2003–2006, including 8504 participants—639 in the asthma group and 7865 in the non-asthma group [35]. Serum vitamin C levels, measured using HPLC, were available for both groups. After sample weighting, significant differences were observed in family asthma history, ethnicity, and gender between the groups (*p* < 0.05). At the same time, serum vitamin C levels were not statistically different between groups (*p* > 0.05).

Conversely, a different study focusing on patients with a prior asthma diagnosis found that patients with severe asthma had significantly lower plasma ASC levels than patients with mild-to-moderate asthma and healthy subjects [36]. This study assessed dietary antioxidant intake and asthma severity using questionnaires. In addition, the study evaluated plasma concentrations of ASC, vitamin E, carotenoids, bilirubin, albumin, uric acid, and total antioxidant status in 53 patients with mild-to-moderate asthma, 28 severe asthmatic patients, and 43 healthy subjects. Intake levels of vitamins C, E, A, and carotene were superimposable among groups. However, plasma ASC levels were lower in patients with severe asthma than in mild-to-moderate asthmatics and control subjects.

## 7. Asthma and Ascorbic Acid Supplementation

Many studies have investigated the effects of vitamin C supplementation in patients with type 2 asthma. For example, a multicentre, prospective, observational study analyzed 71 patients with allergy-related diseases (30 with AR and 10 with asthma) with a diagnosed vitamin C deficiency [40]. These patients received intravenous treatment with 7.5 g of vitamin C diluted in a 0.9% NaCl solution to counteract oxidative stress-related depletion. The treatment lasted 2–3 weeks for those with acute deficiency and 11–12 weeks for those with chronic deficiency. Before starting vitamin C supplementation, the most commonly reported symptoms were pruritus (n = 31), rhinitis (n = 26), and restlessness (n = 15), which were monitored throughout the study. During the observation period, 97.1% of patients experienced symptom improvement, with the mean disease-specific symptom score significantly decreasing at the final visit (*p* < 0.0001).

A review published in 2014 investigated the effect of vitamin C supplementation on exercise-induced bronchoconstriction (EIB). They compared three different trials and concluded that supplementation of 0.5 to 2 g/day vitamin C halved the postexercise FEV_1_ decline by 48% (95% CI: 33% to 64%) [56].

This outcome could be explained by a proven Vitamin C dose-dependent relaxant effect on tracheal smooth muscle through mechanisms such as β-adrenergic receptor stimulation and cyclooxygenase pathway inhibition. Deficiency in vitamin C also seemed to increase bronchoconstrictor PGF2α levels and reduce PGE2, which has bronchodilatory properties. Nitric oxide (NO) is another factor implicated in EIB, and vitamin C supplementation has been reported to decrease NO levels in EIB patients [41]. Notably, a single oral dose of vitamin C induces a rapid increase in mucosal vitamin C levels. This outcome suggests that even short-term supplementation may help protect the airways against acute oxidative stress [57,58]. These findings suggest that ASC can mitigate EIB and airway inflammation in asthmatic patients. A randomized, placebo-controlled, double-blind crossover trial examined the effects of ASC supplementation (1.5 g/day of ASC for two weeks or a placebo) on EIB in eight asthmatic subjects [59]. Pulmonary function, symptoms of asthma, FeNO, and urinary leukotriene (LTC4–E4) and prostaglandin (9α,11β-PGF2) levels were measured at baseline and after each treatment period. ASC supplementation significantly reduced the maximum post-exercise FEV_1_ decline. Additionally, post-exercise FeNO, LTC4–E4, and 9α,11β-PGF2 levels were significantly lower in the ASC group.

Studies in animals, including guinea pigs and mice, demonstrated that vitamin C reduced airway resistance and bronchoconstriction caused by stimuli like histamine and methacholine [60,61]. However, these experimental animal studies have to be considered theoretical as they need confirmation in humans. In this regard, vitamin C deficiency increases airway hyperresponsiveness, further emphasizing its role in managing respiratory disorders [13]. These findings may suggest that vitamin C could reduce airway obstruction in asthma and some other related diseases, like chronic obstructive pulmonary disease (COPD), lung infection, and lung cancer.

## 8. Atopic Dermatitis and Ascorbic Acid

The pathogenesis of atopic dermatitis (AD) is characterized by an imbalance in immune responses, with a predominant activation of the Th2 inflammatory pathway. This leads to increased production of cytokines such as IL-4, IL-5, and IL-13, which contribute to skin barrier dysfunction, type 2 inflammation, and IgE-mediated hypersensitivity.

The use of ASC derivatives in treating AD has shown great promise, as demonstrated by some recent studies [62,63]. In particular, cutaneous administration of DDH-1, an ASC derivative that is more stabilized and with better skin penetrability, tested using an AD mouse model, seemed to significantly reduce the expression of pro-inflammatory cytokines like TNF-α, IL-1β, and IL-6 in the skin lesion. In this study, DDH-1 administration also considerably diminished the Th2-associated chemokine expression (like CCL17 and CCL22 levels), conveying the idea that ASC may be used in AD treatment for reducing the doses and adverse effects of corticosteroids and also as a monotherapy for AD prevention [62].

In addition, AD is also characterized by structural and functional impairment of the protective skin layer. Keratinocytes and their intercellular lipids play a crucial role in maintaining the structural integrity of the human skin barrier. Vitamin C has been shown to have additional beneficial effects by promoting keratinocyte differentiation and the synthesis of intercellular material. Specifically, Vitamin C stimulates ceramide production in keratinocytes, the most prevalent lipids in skin barrier structures, thereby enhancing the overall function of the outer skin defense [37,64].

There is no equally encouraging evidence of effectiveness regarding oral vitamin C supplementation for preventing or treating AD.

A study involving 180 children with AD and 242 without AD assessed dietary intake using a modified food frequency questionnaire (FFQ) [65]. The intake of nutrients with antioxidant activity, including folic acid, iron, vitamins A, C, and E, was evaluated from diet and supplements. In the same group of patients, fasting blood samples were collected to analyze fat-soluble vitamins (retinol, α-tocopherol, and β-carotene), vitamin C, and total IgE concentrations. After adjusting for confounders, higher dietary intakes of vitamin E, β-carotene, iron, and folic acid were associated with a lower risk of AD. However, vitamin C intake and micronutrient supplementation (mean 50 mg/day of vitamin C) showed no significant association with AD risk. Higher serum α-tocopherol and retinol levels were linked to a reduced risk of AD, while serum β-carotene suggested a protective effect with marginal significance. Plasma vitamin C levels showed no significant difference between children with and without AD. Similarly, no association has been demonstrated between dietary intakes of vitamins E or C during pregnancy and AD in the second year of a baby’s life [64].

## 9. Conclusions

Allergic diseases, such as asthma, AR, and AD, are driven by type 2 inflammation, with oxidative stress exacerbating the inflammatory process. In asthma, elevated levels of FeNO serve as a biomarker of disease activity, indicating increased NO production that contributes to airway inflammation and exacerbates symptoms.

Vitamin C, recognized as a potent antioxidant, has garnered attention for its ability to mitigate oxidative stress, a hallmark of inflammatory diseases like asthma. As a powerful antioxidant, it can counteract oxidative stress by neutralizing ROS and modulating inflammatory pathways, as reported above. It has been shown to lower NO levels by reducing iNOS activity, which could reduce inflammation and improve asthma control. By reducing oxidative stress, Vitamin C mitigates the harmful effects of ROS, supports immune function, and improves overall immune balance, which is critical in managing asthma and other allergic conditions.

Several studies have demonstrated that vitamin C supplementation helps reduce oxidative stress in the airways, making it a potential therapeutic option for patients with asthma. The evidence suggests that regular vitamin C intake through diet or pharmacological supplementation could help manage the underlying inflammation that drives asthma, making it an important factor to consider in asthma care.

Given the promising results from studies involving more severe asthma cases, vitamin C could serve as a valuable adjunct therapy, particularly for individuals who experience persistent symptoms and elevated FeNo levels despite standard treatment.

However, it is essential to note that while studies have shown promising results in asthma patients, the effects of vitamin C supplementation on AR remain inconclusive. For instance, studies involving vitamin C supplementation in individuals with AR have failed to show a significant benefit in reducing nasal responses to allergens. This fact suggests that while vitamin C may play a role in reducing systemic inflammation, its direct impact on rhinitis symptoms might be less pronounced or require a more targeted approach. For example, there are no targeted clinical studies on the topical use of ASC supplementation in patients with uncontrolled allergic rhinitis. Therefore, vitamin C has equally high potential if we consider the common pathogenic mechanisms underlying both AR and asthma; however, in the analyzed studies, we have reported better results on asthma control rather than improving AR-specific symptoms. Targeted studies with larger patient samples are needed to understand clinical efficacy better.

Similarly, vitamin C’s role in AD is still under investigation. A combination of immune system dysregulation and oxidative stress drives the skin barrier dysfunction that characterizes AD. Some studies have shown that vitamin C can promote keratinocyte differentiation and enhance the production of intercellular lipids, which are essential for maintaining the skin’s protective barrier. ASC derivatives have been shown to reduce pro-inflammatory cytokine expression and serve as a valuable treatment option for reducing inflammation in AD. However, studies investigating the role of oral vitamin C supplementation in preventing or treating AD have not provided clear evidence of its effectiveness. While specific studies have reported no significant association between dietary vitamin C intake and AD risk, the potential for localized or topical vitamin C therapies remains an area of interest in managing skin-related allergic conditions.

In conclusion, the divergent results between AR, asthma, and AD could highlight the complexity of vitamin C’s role in immune modulation and inflammation. While current evidence supports the potential role of vitamin C as an antioxidant in reducing oxidative stress, particularly in asthma, further investigation is needed to explore the optimal dosage and duration of supplementation to understand its therapeutic potential fully. Larger, well-designed clinical trials are essential to determine whether a sufficient vitamin C intake, through regular diet intake or supplementation, can significantly impact asthma management, particularly in reducing inflammation and improving airway function. In these studies, FeNO should be considered a valuable marker for monitoring bronchial inflammation levels, helping, for example, to assess the effectiveness of the ASC supplementation in reducing oxidative stress and managing airway inflammation over time.

Moreover, exploring the potential benefits of vitamin C supplementation in other allergic conditions, such as AR and AD, would further illuminate its broader therapeutic effects. Ultimately, ongoing studies are crucial to establish concrete recommendations for using vitamin C to treat and prevent asthma and other allergic diseases.

## Figures and Tables

**Table 1:** In vivo studies: Vitamin C intake and allergy.

Reference	Authors, Years	Type of Study	Number of Patients and Characteristics	Type of Intervention	Effects Described
[30]	Forastiere et al., 2000	O	18,737 Italian children aged 6–7 years	Observation of the effects of fresh fruit consumption on respiratory symptoms using standardized respiratory questionnaires filled in by parents.	Higher fruit consumption (5–7 times/week) reduces the risk of wheezing. Even 1–2 times/week offered some protective effects. This finding was especially noticeable in children with a history of asthma.
[31]	Zhang et al., 2024	O	4715 children and adolescents aged 2–17 years	Observation of the relationship between dietary intake of various vitamins and their supplements (including vitamins A, C, D, E, B1, B2, B6, B12, K, niacin, folic acid, and choline) and childhood asthma. The data were extracted from the NHANES database.	The odds of childhood asthma decreased with elevated vitamin C intake.
[32]	Siripornpanich et al., 2022	O	76 asthmatic children aged 7–17 years	Measured PGF2α concentrations, a marker of oxidative stress, measured plasma levels of zinc, vitamin C, and vitamin E, and correlation with altered pulmonary function.	A total of 72 participants with high oxidative stress. All patients had zinc deficiency, and 40% of them were deficient in vitamin C, a condition linked to more severe asthma and airway obstruction.
[33]	García-García et al., 2023	O	6293 adults aged 20–49 year	Investigate the associations of vitamin A and C intake with asthma and AR, using information from KNHANES.	Higher vitamin C intake (≥75 mg) was significantly associated with a lower prevalence of asthma in participants with high hs-CRP. Vitamin C intake was not associated with AR.
[34]	Garcia-Larsen et al., 2017	O	3206 European adults, 22.8% with asthma symptoms and 19.5% with CRS	Observation of the association between asthma and CRS with intake of fruits and vegetables using information from the GA2LEN screening survey and FFQ.	No consistent evidence was found linking fruit and vegetable intake with asthma or CRS.
[35]	Wang et al., 2024	O	8504 adults, including 639 with asthma and 7865 without asthma	Examine the connection between adult asthma and serum vitamin C levels using a multivariate logistic regression model.	After sample weighting, serum vitamin C was not associated with adult asthma risk (OR = 0.829, 95% CI: 0.660~1.042, *p* 0.104).
[36]	Misso et al., 2005	O	53 mild-to-moderate and 28 severe asthmatic patients and 43 non-asthmatic subjects	Determine whether lower antioxidant intake and plasma antioxidant concentrations are associated with more severe asthma.	Plasma AA was lower in severe (31.9 ± 3.6 microM) compared with mild-to-moderate asthmatic (52.3 ± 2.6) or control subjects (52.7 ± 2.9).
[37]	Oh et al., 2010	O	180 children with AD and 242 without AD	To investigate the association of antioxidant nutritional status with the risk of AD. Diet was assessed using a validated semi-quantitative FFQ. Fasting blood samples were used to analyze fat-soluble vitamins (retinol, alpha-tocopherol, and beta-carotene) and vitamin C.	Vitamin C intake and micronutrient supplementation (mean 50 mg/day) showed no significant association with AD risk.

Abbreviations: O: observational; hs-CRP: serum high-sensitivity C-reactive protein; NHANES: National Health and Nutrition Examination Survey; PGF2α: plasma 8-iso-prostaglandin F2α; KNHANES: Korea National Health and Nutrition Examination Survey; CRS: chronic rhinosinusitis; AR: allergic rhinitis; GA2LEN: Global Allergy and Asthma Network of Excellence; FFQ: food frequency questionnaire; OR: odds ratio; CI: confidence interval; AA: ascorbic acid; AD: atopic dermatitis.

**Table 2:** In vivo studies: Vitamin C supplementation and allergy.

Reference	Authors, Years	Type of Study	Number of Patients and Characteristics	Type of Intervention	Effects Described
[38]	Fortner et al., 1982	RCDB	8 adults with AR	A total of 2 g/day of ascorbic acid or placebo for 4 days.	No difference in the nasal response to the instillation of allergen.
[39]	Tongtako et al., 2018	RCT	27 adults with AR	Three groups: a control group, an exercise group (walking or running for 30 min per session, three times per week for 8 weeks), and a group combining exercise with vitamin C supplementation (2 g/day).	Aerobic exercise significantly improves AR symptoms, with no change with vitamin C supplementation.
[40]	Vollbracht et al., 2018	O	71 patients with allergy-related diseases (30 with AR and 10 with asthma) with a diagnosed vitamin C deficiency	The patients received IV treatment with 7.5 g of vitamin C diluted in a 0.9% NaCl solution for 2–3 weeks for acute deficiency and 11–12 weeks for chronic deficiency.	In total, 97.1% of patients registered symptom improvement, with the mean disease-specific symptom (pruritus, rhinitis, or restlessness) score significantly decreasing at the final visit (*p* < 0.0001).
[41]	Tecklenburg et al., 2007	RCBD	8 asthmatic adults with documented EIB	The patients received either 2 weeks of ascorbic acid supplementation (1.5 g/day) or placebo, followed by a 1-week washout period before switching to the alternative diet. Pre- and post-exercise pulmonary function, asthma symptom scores, and FENO were assessed at the beginning of the trial (usual diet) and at the end of each treatment period.	The ascorbic acid diet significantly reduced (*p* < 0.05) the maximum fall in post-exercise FEV1 compared to the usual and placebo diet. Asthma symptom scores significantly improved, and Post-exercise FENO was significantly lower (*p* < 0.05) on the ascorbic acid diet compared to the placebo and usual diet.

Abbreviations: RCDB: randomized controlled double blind; AR: allergic rhinitis; RCT: randomized controlled trial; O: observational; IV: intravenous; EIB: exercise-induced bronchoconstriction; FENO: fraction of exhaled nitric oxide; FEV1: Forced Expiratory Volume in the first second.

## Data Availability

No new data were created.

## References

[1] Padayatty S.J., Levine M. (2016). Vitamin C: The known and the unknown and Goldilocks. Oral Dis..

[2] Kaźmierczak-Barańska J., Boguszewska K., Adamus-Grabicka A., Karwowski B.T. (2020). Two faces of Vitamin C—Antioxidative and pro-oxidative agent. Nutrients.

[3] Chan S., Xiong P., Zhao M., Zhang S., Zheng R., Ye J., Chan K., Li C., Zhong Z. (2024). Anti-inflammatory effects of natural products from vitamin C-rich fruits. Food Front..

[4] Zhou Y., Phan A.D.T., Akter S., Bobasa E.M., Seididamyeh M., Sivakumar D., Sultanbawa Y. (2023). Bioactive Properties of Kakadu Plum-Blended Products. Molecules.

[5] Cunha-Santos E.C.E., Viganó J., Neves D.A., Martínez J., Godoy H.T. (2018). Vitamin C in camu-camu [Myrciaria dubia (H.B.K.) McVaugh]: Evaluation of extraction and analytical methods. Food Res. Int..

[6] Cefali L.C., de Oliveira Maia L., Stahlschimidt R., Ataide J.A., Tambourgi E.B., Rosa P.C.P., Mazzola P.G. (2018). Vitamin C in Acerola and Red Plum Extracts: Quantification via HPLC, in Vitro Antioxidant Activity, and Stability of their Gel and Emulsion Formulations. J. AOAC Int..

[7] Tsukaguchi H., Tokui T., Mackenzie B., Berger U.V., Chen X.Z., Wang Y., Brubaker R.F., Hediger M.A. (1999). A family of mammalian Na+-dependent L-ascorbic acid transporters. Nature.

[8] Lykkesfeldt J., Tveden-Nyborg P. (2019). The pharmacokinetics of vitamin C. Nutrients.

[9] Levine M., Conry-Cantilena C., Wang Y., Welch R.W., Washko P.W., Dhariwal K.R., Park J.B., Lazarev A., Graumlich J.F., King J. (1996). Vitamin C pharmacokinetics in healthy volunteers: Evidence for a recommended dietary allowance. Proc. Natl. Acad. Sci. USA.

[10] Carr A.C., Maggini S. (2017). Vitamin C and immune function. Nutrients.

[11] Schleicher R.L., Carroll M.D., Ford E.S., Lacher D.A. (2009). Serum vitamin C and the prevalence of vitamin C deficiency in the United States: 2003-2004 National Health and Nutrition Examination Survey (NHANES). Am. J. Clin. Nutr..

[12] Cerullo G., Negro M., Parimbelli M., Pecoraro M., Perna S., Liguori G., Rondanelli M., Cena H., D’Antona G. (2020). The Long History of Vitamin C: From Prevention of the Common Cold to Potential Aid in the Treatment of COVID-19. Front. Immunol..

[13] Martin A. (2001). Nutritional recommendations for the French population: The “apports nutritionnels conseillés” (ANCs). Sci. Aliments.

[14] Ghalibaf M.H.E., Kianian F., Beigoli S., Behrouz S., Marefati N., Boskabady M., Boskabady M.H. (2023). The effects of vitamin C on respiratory, allergic and immunological diseases: An experimental and clinical-based review. Inflammopharmacology.

[15] Global Burden of Disease 2016 Disease and Injury Incidenceand Prevalence Collaborators (2016). Global, regional, and national in -cidence, prevalence, and years lived with disability for 328 dis-eases and injuries for 195 countries, 1990–2016: A systematicanalysis for the Global Burden of Disease Study 2016. Lancet.

[16] Agache I., Akdis C.A., Akdis M., Canonica G.W., Casale T., Chivato T., Corren J., Chu D.K., Del Giacco S., Eiwegger T. (2021). EAACI Biologicals Guidelines-Recommendations for severe asthma. Allergy.

[17] Mims J.W. (2015). Asthma: Definitions and pathophysiology. Int. Forum Allergy Rhinol..

[18] Siddiqui Z.A., Walker A., Pirwani M.M., Tahiri M., Syed I. (2022). Allergic rhinitis: Diagnosis and management. Br. J. Hosp. Med..

[19] Wise S.K., Damask C., Roland L.T., Ebert C., Levy J.M., Lin S., Luong A., Rodriguez K., Sedaghat A.R., Toskala E. (2023). International consensus statement on allergy and rhinology: Allergic rhinitis—2023. Int. Forum Allergy Rhinol..

[20] Mortz C.G., Andersen K.E., Dellgren C., Barington T., Bindslev-Jensen C. (2015). Atopic dermatitis from adolescence to adulthood Theme issue: Atopic dermatitis in the TOACS cohort: Prevalence, persistence and comorbidities. Allergy.

[21] Drucker A.M. (2017). Atopic dermatitis: Burden of illness, quality of life, and associated complications. Allergy Asthma Proc..

[22] Njus D., Kelley P.M., Tu Y.J., Schlegel H.B. (2020). Ascorbic acid: The chemistry underlying its antioxidant properties. Free Radic. Biol. Med..

[23] Mohammed B.M., Fisher B.J., Kraskauskas D., Farkas D., Brophy D.F., Fowler A.A., Natarajan R. (2013). Vitamin C: A novel regulator of neutrophil extracellular trap formation. Nutrients.

[24] Conklin P.L., Foyer C.H., Hancock R.D., Ishikawa T., Smirnoff N. (2024). Ascorbic acid metabolism and functions. J. Exp. Bot..

[25] Lykkesfeldt J., Carr A.C., Tveden-Nyborg P. (2025). The pharmacology of vitamin C. Pharmacol. Rev..

[26] Gasmi A., Shanaida M., Oleshchuk O., Semenova Y., Mujawdiya P.K., Ivankiv Y., Pokryshko O., Noor S., Piscopo S., Adamiv S. (2023). Natural Ingredients to Improve Immunity. Pharmaceuticals.

[27] Sharma P., Raghavan S.A., Saini R., Dikshit M. (2004). Ascorbate-mediated enhancement of reactive oxygen species generation from polymorphonuclear leukocytes: Modulatory effect of nitric oxide. J. Leukoc. Biol..

[28] Caritá A.C., Fonseca-Santos B., Shultz J.D., Michniak-Kohn B., Chorilli M., Leonardi G.R. (2020). Vitamin C: One compound, several uses. Advances for delivery, efficiency and stability. Nanomed. Nanotechnol. Biol. Med..

[29] Micillo E., Bianco A., D’Auria D., Mazzarella G., Abbate G.F. (2000). Respiratory infections and asthma. Allergy.

[30] Forastiere F., Pistelli R., Sestini P., Fortes C., Renzoni E., Rusconi F., Dell’Orco V., Ciccone G., Bisanti L. (2000). Consumption of fresh fruit rich in vitamin C and wheezing symptoms in children. Thorax.

[31] Zhang L., Xu Y., Li X., Yang F., Wang C., Yu C. (2024). Multivitamin consumption and childhood asthma: A cross-sectional study of the NHANES database. BMC Pediatr..

[32] Siripornpanich S., Chongviriyaphan N., Manuyakorn W., Matangkasombut P. (2022). Zinc and vitamin C deficiencies associate with poor pulmonary function in children with persistent asthma. Asian Pac. J. Allergy Immunol..

[33] García-García C., Kim M., Baik I. (2023). Associations of dietary vitamin A and C intake with asthma, allergic rhinitis, and allergic respiratory diseases. Nutr. Res. Pract..

[34] Garcia-Larsen V., Arthur R., Potts J.F., Howarth P.H., Ahlström M., Haahtela T., Loureiro C., Bom A.T., Brożek G., Makowska J. (2017). Is fruit and vegetable intake associated with asthma or chronic rhino-sinusitis in European adults? Results from the Global Allergy and Asthma Network of Excellence (GA2LEN) Survey. Clin. Transl. Allergy.

[35] Wang K., Zhao L., Luo H., Deng C., Gong L., Chen Z. (2024). Association of serum vitamin C levels with Asthma in adults: Results of NHANES 2003–2006 and mendelian randomization study. BMC Pulm. Med..

[36] Misso N.L.A., Brooks-Wildhaber J., Ray S., Vally H., Thompson P.J. (2005). Plasma concentrations of dietary and nondietary antioxidants are low in severe asthma. Eur. Respir. J..

[37] Oh S.Y., Chung J., Kim M.K., Kwon S.O., Cho B.H. (2010). Antioxidant nutrient intakes and corresponding biomarkers associated with the risk of atopic dermatitis in young children. Eur. J. Clin. Nutr..

[38] Fortner B.R., Danziger R.E., Rabinowitz P.S., Nelson H.S. (1982). The effect of ascorbic acid on cutaneous and nasal response to histamine and allergen. J. Allergy Clin. Immunol..

[39] Tongtako W., Klaewsongkram J., Mickleborough T.D., Suksom D. (2018). Effects of aerobic exercise and vitamin C supplementation on rhinitis symptoms in allergic rhinitis patients. Asian Pac. J. Allergy Immunol..

[40] Vollbracht C., Raithel M., Krick B., Kraft K., Hagel A.F. (2018). Intravenous vitamin C in the treatment of allergies: An interim subgroup analysis of a long-term observational study. J. Int. Med. Res..

[41] Tecklenburg S.L., Mickleborough T.D., Fly A.D., Bai Y., Stager J.M. (2007). Ascorbic acid supplementation attenuates exercise-induced bronchoconstriction in patients with asthma. Respir. Med..

[42] Schwarze J., Johnston S.L. (2004). Unravelling synergistic immune interactions between respiratory virus infections and allergic airway inflammation. Clin. Exp. Allergy..

[43] Bhoot H.R., Zamwar U.M., Chakole S., Anjankar A. (2023). Dietary Sources, Bioavailability, and Functions of Ascorbic Acid (Vitamin C) and Its Role in the Common Cold, Tissue Healing, and Iron Metabolism. Cureus.

[44] Miller R.L., Grayson M.H., Strothman K. (2021). Advances in asthma: New understandings of asthma’s natural history, risk factors, underlying mechanisms, and clinical management. J. Allergy Clin. Immunol..

[45] Doss A.M.A., Stokes J.R. (2022). Viral Infections and Wheezing in Preschool Children. Immunol. Allergy Clin..

[46] Bove P.F., van der Vliet A. (2006). Nitric oxide and reactive nitrogen species in airway epithelial signaling and inflammation. Free Radic. Biol. Med..

[47] Van Muylem A., Malinovschi A., Haccuria A., Michils A. (2020). Exhaled nitric oxide and its predictive power related to lung function and bronchial inflammation. Biochem. Pharmacol..

[48] Biedrzycki G., Wolszczak-Biedrzycka B., Dorf J., Maciejczyk M. (2024). The antioxidant barrier, oxidative/nitrosative stress, and protein glycation in allergy: From basic research to clinical practice. Front. Immunol..

[49] Licari A., Castagnoli R., Brambilla I., Marseglia A., Tosca M.A., Marseglia G.L., Ciprandi G. (2018). Asthma endotyping and biomarkers in childhood asthma. Pediatr. Allergy Immunol. Pulmonol..

[50] McKeever T.M., Lewis S.A., Smit H., Burney P., Britton J., Cassano P.A. (2004). Serum nutrient markers and skin prick testing using data from the Third National Health and Nutrition Examination Survey. J. Allergy Clin. Immunol..

[51] Kompauer I., Heinrich J., Wolfram G., Linseisen J. (2006). Association of carotenoids, tocopherols and vitamin C in plasma with AGTOPIand allergic sensitization in adults. Allergo J..

[52] Nakamura K., Wada K., Sahashi Y., Tamai Y., Tsuji M., Watanabe K., Ohtsuchi S., Ando K., Nagata C. (2013). Associations of intake of antioxidant vitamins and fatty acids with asthma in pre-school children. Public Health Nutr..

[53] Sackesen C., Ercan H., Dizdar E., Soyer O., Gumus P., Tosun B.N., Büyüktuncer Z., Karabulut E., Besler T., Kalayci O. (2008). A comprehensive evaluation of the enzymatic and nonenzymatic antioxidant systems in childhood asthma. J. Allergy Clin. Immunol..

[54] Shanklin D.R., O’Dell T.E. (1966). Ascorbic acid and the lung. Nature.

[55] Larsson N., Rankin G.D., Bicer E.M., Roos-Engstrand E., Pourazar J., Blomberg A., Mudway I.S., Behndig A.F. (2015). Identification of vitamin C transporters in the human airways: A cross-sectional in vivo study. BMJ Open.

[56] Hemilä H. (2014). The effect of vitamin C on bronchoconstriction and respiratory symptoms caused by exercise: A review and statistical analysis. Allergy Asthma Clin. Immunol..

[57] Schock B.C., Koostra J., Kwack S., Hackman R.M., Van Der Vliet A., Cross C.E. (2004). Ascorbic acid in nasal and tracheobronchial airway lining fluids. Free Radic. Biol. Med..

[58] Behndig A.F., Blomberg A., Helleday R., Kelly F.J., Mudway I.S. (2009). Augmentation of respiratory tract lining fluid ascorbate concentrations through supplementation with vitamin C. Inhal. Toxicol..

[59] Jeong Y.J., Kim J.H., Kang J.S., Lee W.J., Hwang Y.I. (2010). Mega-dose vitamin C attenuated lung inflammation in mouse asthma model. Anat. Cell Biol..

[60] Sipahi E., Ercan Z.S. (1997). The mechanism of the relaxing effect of ascorbic acid in guinea pig isolated tracheal muscle. Gen. Pharmacol..

[61] Kitahata K., Matsuo K., Sato M., Susami Y., Hara Y., Morikawa T., Oiso N., Kawada A., Otsuka A., Nakayama T. (2022). Anti-allergic effect of ascorbic acid derivative DDH-1 in a mouse model of atopic dermatitis. Exp. Dermatol..

[62] Chiu Y.H., Wu Y.W., Hung J.I., Chen M.C. (2021). Epigallocatechin gallate/L-ascorbic acid-loaded poly-γ-glutamate microneedles with antioxidant, anti-inflammatory, and immunomodulatory effects for the treatment of atopic dermatitis. Acta Biomater..

[63] Wang K., Jiang H., Li W., Qiang M., Dong T., Li H. (2018). Role of vitamin C in skin diseases. Front. Physiol..

[64] Kim J., Yun H., Cho Y. (2011). Analysis of ceramide metabolites in differentiating epidermal keratinocytes treated with calcium or vitamin C. Nutr. Res. Pract..

[65] Martindale S., McNeill G., Devereux G., Campbell D., Russell G., Seaton A. (2005). Antioxidant intake in pregnancy in relation to wheeze and eczema in the first two years of life. Am. J. Respir. Crit. Care Med..

